# Supplementary material for: Heat adaptation of phage T7 under an extended genetic code
Source: Virus Evol. 2021 Dec 1;7(2):veab100. doi: 10.1093/ve/veab100 (PMC8923235; doi:10.1093/ve/veab100)
Supplement: veab100_Supp [file veab100_supp.zip › Sequencing_Analysis.zip!/Sequencing_Analysis/A2/evidence/JC_47.html]

BRESEQ :: Evidence


| New junction evidence | | | | | | | | | | | |
| --- | --- | --- | --- | --- | --- | --- | --- | --- | --- | --- | --- |
|  | | seq id | position | reads (cov) | reads (cov) | score | skew | freq | annotation | gene | product |
| \* | ? | NC\_001604.1 | = 7781 | 452 (0.990) | 10 (0.020) | 7/558 | 19.9 | 2.3% | intergenic (–/–) | *–/–* | –/– |
|  | ? | NC\_001604.1 | 7899 = | 446 (1.050) |  |  |  |  | intergenic (–/–) | *–/–* | –/– |
| Rejected: Coverage evenness skew score above cutoff. | | | | | | | | | | | |
| Rejected: Frequency below/above cutoff threshold. | | | | | | | | | | | |

Only 100 of 122 total aligned reads displayed.  

`TAGCTACATGGAGGAAACACCTGATGGCTCTTTGCGGCACCCATCGTTCGTAATGTTCCGTGGCACCGAGGACAACCCTCAAGAGAAAATGTAATCACACTGGCTCACCTTCGGGTGGGCCTTTCTGCGTTTATAAGGAGACACTTTATGTTTAAGAAGGTTGGTAAATTCCTTGCGGCTTTGGCAGCTATCCTGACGCTTGCGTATATTCTTGCGGTATACCCTCAAGTAGCACTAGTAGTAGTTGGCGCTTGTTACTTAGCGGCAGTGTGTGCTTGCGTGTGGAGTATAGTTAACTGGTAATACGACTCACTAAAGGAG‑‑‑‑‑‑‑‑‑‑‑‑‑‑‑‑‑‑‑‑‑‑‑‑‑‑‑‑‑‑‑‑‑‑‑‑‑‑‑‑‑‑‑‑‑‑‑‑‑‑‑‑‑‑‑‑‑‑‑‑‑‑‑‑‑‑‑‑‑‑‑‑‑‑‑‑‑‑‑‑‑‑‑‑‑‑‑‑‑‑‑‑‑‑‑‑‑‑‑‑‑‑‑‑‑‑‑‑‑‑‑‑‑‑‑‑‑‑‑‑‑‑‑‑‑‑‑‑‑‑‑‑‑‑‑‑‑‑‑‑‑‑‑‑‑‑‑‑‑‑‑‑‑‑‑‑‑‑‑‑‑‑‑‑‑‑‑‑‑‑‑‑‑‑‑‑‑‑‑‑‑‑‑‑‑‑‑‑‑‑‑‑‑‑‑‑‑‑‑‑‑‑‑‑‑‑‑‑‑‑‑‑‑‑‑‑‑‑‑‑‑‑‑‑‑‑‑‑‑‑‑‑‑‑‑‑‑‑‑‑‑‑‑‑‑‑‑‑‑‑‑‑‑‑‑‑‑‑‑‑‑‑‑‑‑‑‑‑‑‑‑‑‑‑‑‑‑‑‑‑‑‑‑‑‑‑‑‑‑‑‑‑‑‑‑‑‑  >  NC_001604.1/7461‑7781`  
`‑‑‑‑‑‑‑‑‑‑‑‑‑‑‑‑‑‑‑‑‑‑‑‑‑‑‑‑‑‑‑‑‑‑‑‑‑‑‑‑‑‑‑‑‑‑‑‑‑‑‑‑‑‑‑‑‑‑‑‑‑‑‑‑‑‑‑‑‑‑‑‑‑‑‑‑‑‑‑‑‑‑‑‑‑‑‑‑‑‑‑‑‑‑‑‑‑‑‑‑‑‑‑‑‑‑‑‑‑‑‑‑‑‑‑‑‑‑‑‑‑‑‑‑‑‑‑‑‑‑‑‑‑‑‑‑‑‑‑‑‑‑‑‑‑‑‑‑‑‑‑‑‑‑‑‑‑‑‑‑‑‑‑‑‑‑‑‑‑‑‑‑‑‑‑‑‑‑‑‑‑‑‑‑‑‑‑‑‑‑‑‑‑‑‑‑‑‑‑‑‑‑‑‑‑‑‑‑‑‑‑‑‑‑‑‑‑‑‑‑‑‑‑‑‑‑‑‑‑‑‑‑‑‑‑‑‑‑‑‑‑‑‑‑‑‑‑‑‑‑‑‑‑‑‑‑‑‑‑‑‑‑‑‑‑‑‑‑‑‑‑‑‑‑‑‑‑‑‑‑‑‑‑‑‑‑‑‑‑‑‑‑‑‑‑‑‑‑‑‑taatacgactcactaaaggagACACTATATGTTTCGACTTCATTACAACAAAAGCGTTAAGAATTTCACGGTTCGCCGTGCTGACCGTTCAATCGTATGTGCGAGCGAGCGCCGAGCTAAGATACCTCTTATTGGTAACACAGTTCCTTTGGCACCGAGCGTCCACATCATTATCACCCGTGGTGACTTTGAGAAAGCAATAGACAAGAAACGTCCGGTTCTTAGTGTGGCAGTGACCCGCTTCCCGTTCGTCCGTCTGTTACTCAAACGAATCAAGGAGGTGTTCTGATGGGACTGTTAGATGGTGAAGCCTGGGAA  >  NC_001604.1/7899‑8195`  

                                                                                                                                                                                                                                                                                                                                                                                                                                                                                                                                                                                                                                            
`gacagACATGGAGGAAACACCTGATGGCTCTTTGCGGCACCCATCGTTCGTAATGTTCCGTGGCACCGAGGACAACCCTCAAGAGAAAATGTAATCACACTGGCTCACCTTCTGGTGGGCCTTTCTGCGTTTATAAGGAGACACTTTATGTTTAAGAAGGTTGGTAAATTCCTTGCGGCTTTGGCAGCTATCCTTACGCTTGCGTATATTCTTGTGGTATACCCTCAAGTAGCACTAGTAGTAGTTGGCGCTTGTTACTTAGCGGCAGTGTGTGCTTGCGTGTGGAGTATAGTTAACTGGT                                                                                                                                                                                                                                                                                                                               <  1:312042/296‑1`  
  `GCTACATGGAGGAAACACCTGATGGCTCTTTGCGGCACCCATCGTTCGTAATGTTCCGTGGCACCGAGGACAACCCTCAAGAGAAAATGTAATCACACTGGCTCACCTTCGGGTGGGCCTTTCTGCGTTTATAAGGAGACACTTTATGTTTAAGAAGGTTGGTAAATTCCATGCGGCTTTGGCAGCTATCCTGACGCTTGCGTATATTCTTGTGGTATACCCTCAAGTAGCACTAGTAGTAGTTGGCGCTTGTTACTTAGCGGCAGTGTGTGCTTGCGTGTGGAGTATAGTTAACTGGTAA                                                                                                                                                                                                                                                                                                                             >  1:391133/1‑301`  
  `GCTACATGGAGGAAACACCTGATGGCTCTTTGCGGCACACATCGTTCGTAATGTTCCGTGGAACCGAGGACAACCCTCAAGAGAAAATGTAATCACACTGGCTCACCTTCGGGTGGGCCTTTCTGCGTTTATAAGGAGACACTTTATGTTTAAGAAGGTTGGTAAATTCCTTGCAGCTTTGGCAGCTATCCGGACGCTTGCGTATATTCTTGCGGTATACCCTCAAGTAGCACTAGTAGTAGTTGGCGCTTGTTACTTAGCGGCAGTGTGTGCTTGCGTGTGGAGTATAGTTAACTGGTAA                                                                                                                                                                                                                                                                                                                             >  1:73959/1‑301`  
     `ACATGGAGGAAACACCTGATTGCTCTTTGCGGCACCCATCGTTCGTAATGTTCCGTGGCACCGAGGACAACCCTCAAGAGAAAATGTAATCACACTGGCTCACCTTCGGGTGGGCCTTTCTGCGTTTATAAGGAGACACTTTATGTTTAAGAAGGTTGGTAAATTCCTTGCGGCTTTGGCAGCTATCCTGACGCTTGCGTATATTCTTGCGGTATACCCTCAAGTAGCACTAGTAGTAGTTGGCGCTTGTTACTTAGCGGCAGTGTGTGCTTGCGTGTGGAGTATAGTTAACTGGTAATAC                                                                                                                                                                                                                                                                                                                          <  1:106629/301‑1`  
     `ACATGGAGGAAACACCTGATGGCTCTTTTCGGCACCCATCGTTCGTAATGTTCCGTGGCACCGAGGACAACCCTCAAGAGAAAATGTAATCACACTGGCTCACCTTCGGGTGGGCCTTTCTGCGTTTATAAGGAGACACTTTATGTTTAAGAAGGTTGGTAAATTCCTTGCGGCTTTGGCAGCTATCCTGACGCTTGCGTATATTCTTGCGGTATACCCTCAAGTAGCACTAGTAGTAGTTGGTGCTTGTTACTTAGCGGCAGTGTGTGCTTGCGTGTGGAGTATAGTTAACTGGTAATAC                                                                                                                                                                                                                                                                                                                          <  1:761800/301‑1`  
     `ACATGGAGGAAACACCTGATGGCTCTTTGCGGCACCCATCGTTCGTAATGTTCCGTGGCACCTAGGACAACCCTCAAGAGAAAATGTAATCACACTGGCTCACCTTCGGGTGGGCCTTTCTGCGTTTATAAGGAGACACTTTATGTTTAAGAAGGTTGGTAAATTCCTTGCGGCTTTGGCAGCTATCCTGACGCTTGCGTATATTCTTGCGGTATACCCTCAAGTAGCACTAGTAGTAGTTGGCGCTTGTTACTTAGCGGCAGTGTGTGCTTGCGTGTGGAGTATAGTTAACTGGTAATAC                                                                                                                                                                                                                                                                                                                          >  1:600766/1‑301`  
     `ACATGGAGGAAACACCTGATGGCTCTTTGCGGCACCCATCGTTCGTAATGTTCCGTGGCACCGAGGACAACCCTCAAGAGAAAATGTAATCACACTGGCTCACCTTCGGGTGGGCCTTTCTGCGTTTATAAGGAGACACTTTATGTTTAAGAAGGTTGGTAAATTCCTTGCGGCTTTGGCAGCTATCCTGACGCTTGCGTATATTCTTGCGGTATACCCTCAAGTAGCACTAGTAGTAGTTGGCGCTTGTTACTTAGCGGCAGTGTGTGCTTGCGTGTGGAGTATAGTTAACTGGTAATAC                                                                                                                                                                                                                                                                                                                          >  1:347139/1‑301`  
     `ACATGGAGGAAACACCTGATGGCTCTTTGCGGCACCCATCGTTCGTAATGTTCCGTGGCACCGAGGACAACCCTCAAGAGAAAATGTAATCACACTGGCTCACCTTCGGGTGGGCCTTTCTGCGTTTATAAGGAGACACTTTATGTTTAAGAAGGTTGGTAAATTCCTTGCGGCTTTGGCAGCTATCCTGACGCTTGCGTATATTCTTGCGGTATACCCTCAAGTAGCACTAGTAGTAGTTGGCGCTTGTTACTTAGCGGCAGTGTGTGCTTGCGTGTGGAGTATAGTTAACTGGTAATAC                                                                                                                                                                                                                                                                                                                          <  1:733409/301‑1`  
      `aATGGAGGAAACACCTGATGGCCCTTTGCGGCACCCATCGTTCGTAATGTTCCGTGGCACCGAGGACAACCCTCAAGAGAAAATGTAATCACACTGGCTCACCTTCGGGTGGGCCTTTCTGCGTTTATAAGGAGACACTTTATGTTTAAGAAGGTTGGTAAATTCCTTGCGGCTTTGGCAGCTATCCTGACGCTTGCGTATATTCTTGCGGTATACCCTCAAGTAGCACTAGTAGTAGTTGGCGCTTGTTACTTAGCGGCAGTGTGTGCTTGCGTGTGGAGTATAGTTAACTGGTAATACG                                                                                                                                                                                                                                                                                                                         <  1:273728/300‑1`  
      `CATGGAGGAAACACCTGATGGCTCTTTGCGGCACCCATCGTTCGTAATGTTCCGTGGCACCGAGGACAACCCTCAAGAGCAAATGTAATCACACTGGCTCACCTTCGGGTGGGCCTTTCTGCGTTTATAAGGAGACACTTTATGTTTAAGAAGGTTGGTTAATTCCTTGCGGCTTTGGCAGCTATCCTGACGCTTGCGTATATTCTTGCGGTATACCCTCAAGTAGCACTACTAGTCGTTGGCGCTTGTTACTTAGCGGCAGTGTGTGCTTGCGTGTGGAGTATAGTTAACTGGTAATACG                                                                                                                                                                                                                                                                                                                         >  1:631508/1‑301`  
      `CATGGAGGAAACACCTGATGGCTCTTTGCGGCACCCATCGTTCGTAATGTTCCGTGGCACCGAGGACAACCCTCAAGAGAAAATGTAATCACACTGGCTCACCTTCGGGTGGGCCTTTCTGCGTTTATAAGGAGACACTTTATGTTTAAGAAGGTTGGTAAATTCATTGCGGCTTTGGCAGCTATCCTGACGCTTGCGTATATTCTTGCGGTATACCCTCAAGTAGCACTAGTAGTAGTTGGAGCTTGTTACTTAGCGGCAGTGTGTGCTTGCGTGTGGAGTATAGTTAACTGGTAATACG                                                                                                                                                                                                                                                                                                                         <  1:624565/301‑1`  
      `CATGGAGGAAACACCTGATGGCTCTTTGCGGCACCCATCGTTCGTAATGTTCAGTGGCACCGAGGACAACCCTCAAGAGAAAATGTAAT‑‑CACTGGCTCACCTTCGGGTGGGCCTTTCTGCGTTTATAAGGAGACACTTTATGTTTAAGAAGGTTGGTAAATTCCTTGCGGCTTTGGCAGCTATCCTGACGCTTGCGTATATTCTTGCGGTATACCCTCAAGTAGCACTAGTAGTAGTTGGCGCTTGTTACTTAGCGGCAGTGTGTGCTTGCGTGTGGAGTATAGTTAACTGGTAATACGAC                                                                                                                                                                                                                                                                                                                       >  1:582041/1‑301`  
       `ATGGAGGAACCACCGGATGGCTCTTTGCGGCACCCATCGTTCGTAATGTTCCGTGGCACCGAGGACAACCCTCAAGAGAAAATGTAATCACACTGGCTCACCTTCGGGTGGGCCTTTCTGCGTTTATAAGGAGACACTTTATGTTTAAGAAGGTTGGTAAATTCCTTGCGGCTTTGGCAGCTATCCTGACGCTTGCGTATATTCTTGCGGTATACCCTCAAGTAGCACTAGTAGTAGTTGGCGCTTGTTACTTAGCGGCAGTGTGTGCTTGCGTGTGGAGTATAGTTAACTGGTAATACGA                                                                                                                                                                                                                                                                                                                        <  1:744161/301‑1`  
        `gGGAGGAAACACCTGATGGCTCTTTGCGGCACCCATCGTTCGTAATGTTCCGTGGCACCGAGGACAACCCTCAAGAGAAAATGTAATCACACTGGCTCACCTTCGGGTGGGCCTTTCTGCGTTTATAAGGAGACACTTTATGTTTAAGAAGGTTGGTAAATTCCTTGCGGCTTTGTCAGCTATCCTGACGCTTGCGTATATTCTTGCGGTATACCCTCAAGTAGCACTAGTAGTAGTTGGCGCTTGTTACTTAGCGGCAGTGTGTGCTTGCGTGTGGAGTATAGTTAACTGGTAATACGAC                                                                                                                                                                                                                                                                                                                       <  1:694199/300‑1`  
          `GAGGAAACACCTGATGTCTCTTTGCGGCACCCATCGTTCGTAATGTTCCGTGGCACCGAGGACAACCCTCAAGAGAAAATGTAATCACACTGGCTCACCTTCGGGTGGGCCTTTCTGCGTTTATAAGGAGACACTTTATGTTTAAGAAGGTTGGTAAATTCCTTGCGGCTTTGGCAGCTATCCTGACGCTTGCGTATATTCTTGCGGTATACCATCAAGTAGCACTAGTAGTAGTTGGCGCTTGTTACTTAGCGGCAGTGTGTGCTTGCGTGTGGAGTATAGTTAACTGGTAATACGACTC                                                                                                                                                                                                                                                                                                                     >  1:165491/1‑301`  
          `GAGGAAACACCTGATGGCTCTTTGCGGCACCCATCGTTCGTAATGTTCCGTGGCACCGAGGACAACCCTCAAGAGAAAATGTAATCACACTGGCTCACCTTCGGGTGGGCCTTTCTGCGTTTATAAGGAGACACTTTATGTTTAAGAAGGTTGGTAAATTCCTTGCGGCTTTGGCAGCTATCCTGACGCTTGCGTATATTCTTGCGGTATACCCTCAAGTAGCACTAGTAGTAGTTGGCGCTTGTTACTTAGCGGAAGTGTGTGCTTGCGTGTGGAGTATAGTTAACTGGTAATACGACTC                                                                                                                                                                                                                                                                                                                     >  1:124508/1‑301`  
          `GAGGAAACACCTGATGGCTCTTTGCGGCACCCATCGTTCGTAATGTTCCGTGGCACCGAGGACAACCCTCAAGAGAAAATGTAATCACACTGGCTCACCTTCGGGTGGGCCTTTCTGCGTTTATAAGGAGACACTTTATGTTTAAGAAGGTTGGTAAATTCCTTGCGGCTTTGGCAGCTATCATGACGCTTGCGTATATTCTTGCGGTATACCATCAAGTAGCACTAGTAGTAGTTGGCGCTTGTTACTTAGCGGCAGTGTGTGCTTGCGTGTGGAGTATAGTTAACTGGTAATACGACTC                                                                                                                                                                                                                                                                                                                     >  1:93923/1‑301`  
           `AGGAAACACCTGATGGCTCTTTGCGGCACCCATCGTTCGTAATGTTCCGTGGCACCGAGGACAACCCTCAAGAGAAAATGTAATCACACTGGCTCACCTTCGGGTGGGCCTTTCTGCGTTTATAAGGAGACACTTTATGTTTAAGAAGGTTGGTAAATTCCTTGCGGCTTTGGCAGCTATCCTGACGCTTGCGTATATTCTTGCGGTATACCCTCAAGTAGCACTAGTAGTATTTGGCGCTTGTTACTTAGCGGCCGTGTGTGCTTGCGTGTGGAGTATAGTTAACTGGTAATACGACTta                                                                                                                                                                                                                                                                                                                    >  1:228587/1‑299`  
                 `CACCTGATGGCTCTTTGCTGCACCCATCGTTCGTAATGTTCCGTGGCACCGAGGACAACCCTCAAGAGAAAATGTAATCACACTGGCTCACCTTCGGGTGGGCCTTTCTGCGTTTATAAGGAGACACTTTATGTTTAAGAAGGTTGGTAAATTCCTTGCGGCTTTGGCAGCTATCCTGACGCTTGCGTATATTCTTGCGGTATACCCTCAAGTAGCACTAGTAGTAGTTGGCGCTTGTTACTTAGCGGCAGTGTGTGCTTGCGTGCGGAGTATAGTTAACTGGTAATACGACTCACTAAAG                                                                                                                                                                                                                                                                                                              >  1:24471/1‑301`  
                 `CACCTGATGGCTCTTTGCGGCACCCATCGTTCGTAATGTTCCGTGGCACCGAGGACAACCCTCAAGAGAAAATGTAATCACACTGGCTCACCTTCTGGTGGGCCTTTCTGCGTTTATAAGGAGACACTTTATGTTTAAGAAGGTTGGTAAATTCCTTGCGGCTTTGGCAGCTATCCTGACGCTTGCGTATATTCTTGCGGTATACCCTCAAGTAGCACTAGTAGTAGTTGGCGCTTGTTACTTAGCGGCAGTGTGTGCTTGCGTGTGGAGTATAGTTAACTGGTAATACGACTCACTAAAG                                                                                                                                                                                                                                                                                                              >  1:106157/1‑301`  
                 `CACCTGATGGCTCTTTGCGGCACCCATCGTTCGTAATGTTCCGTGGCACCGAGGACAACCCTCAAGAGAAAATGTAATCACACTGGCTCACCTTCGGGTGGGCCTTTCTGCGTTTATAAGGAGACACTTTATGTTTAAGAAGGTTGGTAAATTCCTTGCGGCTTTGGCAGCTATCCTGACGCTTGCGTATATTCTTGCGGTATACCCTCAAGTAGCACTAGTAGTAGTTGGCGCTTGTTACTTAGCGGCAGTGTGTGCTTGCGTGTGGAGTATAGTTAACTGGTAATACGACTCACTAAAG                                                                                                                                                                                                                                                                                                              >  1:185197/1‑301`  
                  `gtgggctcggagatgtgtataagagacagTCGTAATGTTCCGTGGCACCGAGGACAACCCTCAAGAGAAAATGTAATCACACTGGCTCACCTTCGGGTGGGCCTTTCTGCGTTTATAAGGAGACACTTTATGTTTAAGAAGGTTGGTAAATTCCTTGCGGCTTTGGCAGCTATCCTGACGCTTGCGTATATTCTTGCGGTATACCCTCAAGTAGCACTAGTAGTAGTTGGCGCTTGTTACTTAGCGGCAGTGTGTGCTTGCGTGTGGAGTATAGTTAACTGGTAATACGACTCACTAAAGG                                                                                                                                                                                                                                                                                                             <  1:127404/272‑1`  
                  `cCCTGATGGCTCTTTGCGGCACCCATCGTTCGTAATGTTCCGTGGCACCGAGGACAACCCTCAAGAGAAAATGTAATCACACTGGCTCACCTTCGGGTGGGCCTTTCTGCGTTTATAAGGAGACACTTTATGTTTAAGAAGGTTGGTAAATTCCTTGCGGCTTTGGCAGCTATCCTGACGCTTGCGTATATTCTTGCGGTATACCCTCAAGTAGCACTAGTAGTAGTTGGCGCTTGTTACTTAGCGGCAGTGTGTGCTTGCGTGTGGAGTATAGTTAACTGGTAATACGACTCACTAAAGG                                                                                                                                                                                                                                                                                                             <  1:465503/300‑1`  
                  `ataagagacagCTTTGTGGCACCCATCGTTCGTAATGTTCCGTGGCACCGAGGACAACCCTCAAGAGAAAATGTAATCACACTGGCTCACCTTCGGGTGGGCCTTTCTGCGTTTATAAGGAGACACTTTATGTTTAAGAAGGTTGGTAAATTCCTTGCGGCTTTGGCAGCTATCCTGACGCTTGCGTATATTCTTGCGGTATACCCTCAAGTAGCACTAGTAGTAGTTGGCGCTTGTTACTTAGCGGCAGTGTGTGCTTGCGTGTGGAGTATAGTTAACTGGTAATACGACTCACTAAAGG                                                                                                                                                                                                                                                                                                             <  1:265832/290‑1`  
                  `ACCTGATGGCTCTTTGCGGCACCCATCGTTCGTAATGTTCCGTGGCACCGAGGACAACCCTCAAGAGAAAATGTAATCACACTGGCTCACCTTCGGGTGGGCCTTTCTGCGTTTATAAGGAGACACTTTATGTTTAAGAAGGTTGGTAAATTCCTTGCGGCTTTGGCAGCTATCCTGACGCTTGCGTATATTCTTGCGGTATACCATCAAGTAGCACTAGTAGTAGTTGGCGCTTGTTACTTAGCGGCAGTGTGTGCTTGCGTGTGGAGTATAGTTAACTGGTAATACGACTCACTAAAGG                                                                                                                                                                                                                                                                                                             <  1:387281/301‑1`  
                  `ACCTGATGGCTCTTTGCGGCACCCATCGTTCGTAATGTTCCGTGGCACCAAGGACAACCCTCAAGAGAAAATGTAATCACACTGGCTCACCTTCGGGTGGGCCTTTCTGCGTTTATAAGGAGACACTTTATGTTTAAGAAGGTTGGTAAATTCCTTGCGGCTTTGGCAGCTATCCTGACGCTTGCGTATATTCTTGCGGTATACCCTCAAGTAGCACTAGTAGTAGTTGGCGCTTGTTACTTAGCGGCAGTGTGTGCTTGCGTGTGGAGTATAGTTAACTGGTAATACGACTAACTAAAGG                                                                                                                                                                                                                                                                                                             <  1:125421/301‑1`  
                   `CCTGATGGCTCTTTGCGGCACCCATCGTTTGTAATGTTCCGTGGCACCGAGTACAACCCTCAAGAGAAAATGTAATCACACTGGCTCACCTTCGGGTGGGCCTTTCTGCGTTTATAAGGAGACACTTTATGTTTAAGAAGGTTGGTAAATTCCTTGCGGCTTTGGCAGCTATCCTGACGCTTGCGTATATTCTTGCGGTATACCCTCAAGTAGCACTAGTAGTAGTTGGCGCTTGTTACTTAGCGGCAGTGTGTGCTTGCGTGTGGAGTATAGTTAACTGGTAATACGACTCACTAAAGGA                                                                                                                                                                                                                                                                                                            >  1:666152/1‑301`  
                   `CCTGATGGCTCTTTGCGGCACCCATCGTTCGTAATGTTCCGTGGCACCGAGGACAACCCTCAAGAGAAAATGTAATCACACTGGCTCACCTTCGGGTGGGCCTTTCTGCGTTTATAAGTAGACACTTTATGTTTAAGAAGGTTGGTAAATTCCTTGCGGCTTTGGCAGCTATCCTGACGCTTGCGTATATTCTTGCGGTATACCCTCAAGTAGCACTAGTAGTAGTTGGCGCTTGTTACGTAGTGGCAGTGTGTGCTTGCGTGTTGAGTATAGTTAACTGGTAATACGACTCACTAAAGGA                                                                                                                                                                                                                                                                                                            >  1:515520/1‑301`  
                   `CCTGATGGCTCTTTGCGGCACCCATCGTTCGTAATGTTCCGTGGCACCGAGGACAACCCTCAAGAGAAAATGTAATCACACTGGCTCACCTTCGGGTGGGCCTTTCTGCGTTTATAAGGAGACACTTTATGTTTAAGAAGGTTGGTAAATTCCTTGCGGCTTTGGCAGCTATCCTGACGCTTTCGTATATTCTTGCGGTATACCCTCAAGTAGCACTAGTAGTAGTTGGCGCTTGTTACTTAGCTGCAGTGTGTGCTTGCGTGTGGAGTATAGTTAACTGGTAATAAGACTCACTAAAGGg                                                                                                                                                                                                                                                                                                            >  1:140199/1‑300`  
                   `CCTGATGGCTCTTTGCGGCACCCATCGTTCGTAATGTTCCGTGGCACCGAGGACAACCCTCAAGAGAAAATGTAATCACACTGGCTCACCTTCGGGTGGGCCTTTCTGCGTTTATAAGGAGACACTTTATGTTTAAGAAGGTTGGTAAATTCCTTGCGGCTTTGGCAGCTATCCTGACGCTTGCGTATATTCTTGCGGTATACCCTCAAGTAGCACTAGTAGTAGTTGGCGCTTGTTACTTAGCGGCAGTGTGTGCTTGCGTGTGGAGTATAGTTAACTGGTAATACGACTCACTAAAGGA                                                                                                                                                                                                                                                                                                            >  1:226616/1‑301`  
                    `CTGATGGCTCTTTGCGGCACCCATCGTTCGTAATGTTCCGTGGCACCGAGGACAACCCTCAAGAGAAAATGTAATCACACTGGCTCACCTTCGGGTGGGCCTTTCTGCGTTTATAAGGAGACACTTTATGTTTAAGAAGGTTGGTAAATTCCTTGCGGCTTTGGCAGCTATCCTGACGCTTGCGTATATTCTTGCGGTATACCCTCAAGTAGCACTAGTAGTAGTTGGCGCTTGTTACTTAGCGGCAGTGTGTGCTTGCGTGTGGAGTATAGTTAACTGGTAATACTACTCACTAAAGGAG                                                                                                                                                                                                                                                                                                           <  1:239257/301‑1`  
                    `CTGATGGCTCTTTGCGGCACCCATCGTTCGTAATGTTCCGTGGCACCGAGGACAACCATCAAGAGAAAATGTAATCACACTGGCTCACCTTCGGGTGGGCCTTTCTGCGTTTATAAGGAGACACTTTATGTTTAAGAAGGTTGGTAAATTCCTTGCGGCTTTAGCAGCTATCCTGACGCTTGCGTATATTCTTGCGGTATACCCTCAAGTAGCACTAGTAGTAGTTGGCGCTTGTTACTTAGCGGCAGTGTGTGCTTGCGTGTGGAGTATAGTTAACTGGTAATACGACTCACTAAAGGAG                                                                                                                                                                                                                                                                                                           >  1:210144/1‑301`  
                                         `CATCGTTCGTAATGTTCCGTGGCACCGAGGACAACCCTCAAGAGAAAATGTAATCACACTGGCTCACCTTCGGGTGGGCCTTTCTGCGTTTATAAGGAGACACTTTATGTTTAAGAAGGTTGGTAAATTCCTTGCGGCTTTGGCAGCTATCCTGACGCTTGCGTATATTCTTGCGGTATACCCTCAAGTAGCACTAGTAGTAGTTGGCGCTTGTTACTTAGCGGCAGTGTGTGCTTGCGTGTGGAGTATAGTTAACTGGTAATACGACTCACTAAAGGctgtctcttatacacatctccga                                                                                                                                                                                                                                                                                      >  1:22207/1‑278`  
                                          `ATCGTTCGTAATGTTCCGTGGCACCGAGGACAACCCTCAAGAGAAAATGTAATCACACTGGCTCACCTTCGGGTGGGCCTTTCTGCGTTTATAAGGAGACACTTTATGTTTAAGAAGATTGGTAAATTCATTGCAGCTTTGGCAGCTATCCTGACGCTTGCGTATATTCTTGCGGTATACCCTCAAGTAGCACTAGTAGTAGTTGGCGCTTGTTACTTAGCGGCAGTGTGTGCTTGCGTGTGGAGTATAGTTAACTGGTAATACGACTCACTAAAGGctgtctcttatacacatctccgag                                                                                                                                                                                                                                                                                     >  1:582769/1‑277`  
                                            `CGTTCGTAATGTTCAGTGGCACCGAGGACAACCCTCAAGAGAAAATGTGATCACACTGGCTCACCTTCGGGTGGGCCTTTCTGCGTTTATAAGGAGACACTTTATGTTTAAGAAGGTTGGTAAATTCCTTGCGGCTTTGGCAGCTATCCTGACGCTTGCGTATATTCTTGCGGTATACCATCAAGTAGCACTAGTAGTAGTTGGCGCTTGTTACTTAGCGGCAGTGTGTGCTTGCGTGTGGAGTATAGTTAACTGGTAATACGACTCACTAAAGGAGctgtctcttatacacatctccgag                                                                                                                                                                                                                                                                                   >  1:787844/1‑277`  
                                                 `GTAATGTTCCGTGGCACCGAGGACAACCCTCAATAGAAAATGTAATCACACTGGCTCACCTTCGGGTGGGCCTTTCTGCGTTTATAAGGAGACACTTTATGTTTAAGAAGGTTGGTAAATTCCTTGCGGCTTTGGCAGCTATCCTGACGCTTGCGTATATTCTTGCGGTATACCCTCAAGTAGCACTAGTAGTAGTTGGCGCTTGTTACTTAGCGGCAGTGTGTGCTTGCGTGTGGAGTATAGTTAACTGGTAATACGACTCACTACAGGAGACACTATATGTTTCGACTTCATTACAACA                                                                                                                                                                                                                                                                              >  1:182601/1‑301`  
                                                 `GTAATGTTCCGTGGCACCGAGGACAACCCTCAATAGAAAATGTAATCACACTGGCTCACCTTCGGGTGGGCCTTTCTGCGTTTATAAGGAGACACTTTATGTTTAAGAAGGTTGGTAAATTCCTTGCGGCTTTGGCAGCTATCCTGACGCTTGCGTATATTCTTGCGGTATACCCTCAAGTAGCACTAGTAGTAGTTGGCGCTTGTTACTTAGCGGCAGTGTGTGCTTGCGTGTGGAGTATAGTTAACTGGTAATACGACTCACTAAAGGAGACACTATATGTTTCGACTTCAGTACAACA                                                                                                                                                                                                                                                                              >  1:182594/1‑301`  
                                                                      `GACAACCCTCAATAGAAAATGTAATCACACTGGCTCACCTTCGGGTGGGCCTTTCTGCGTTTATAAGGAGACACTTTATGTTTAAGAAGGTTGGTAAATTCCTTGCGGCTTTGGCAGCTATCCTGACGCTTGCGTATATTCTTGCGGTATTCCCTCAAGTAGCACTAGTAGTAGTTGGCGCTTGTTACTTAGCGGTAGTGTGTGCTTGCGTGTGGAGTATAGTTAACTGGTAATACGACTCACTAAAGGAGACACTATATGTTTCGACTTCATTACAACAAAAGCGTTAAGAATTTCACGG                                                                                                                                                                                                                                                         <  1:708425/301‑1`  
                                                                                                                                                                                           `agATCCTGACGCTTGCGTATATTCTTGCGGTATACCCTCAAGTAGCACTAGTAGTAGTTGGCGCTTGTTACTTAGCGGCAGTGTGTGCTTGCGTGTGGAGTATAGTTAACTTGTAATACGACTCACTAAATGAGACACTATATGTTTCGACTTCATTACAACAAAAGCGTTAAGAATTTCACGGTTCGCCGTGCTGACCGTTCAATCGTATGTTCGAGCGAGTGCCGAGCTAAGATACCTCTTATTGGTAACACAGTTCCTTTGGCACCGAGCGTCCACATCATTATCACCCATGGTGctg                                                                                                                                    <  1:474594/299‑4`  
                                                                                                                                                                                                 `cggagatgtgtataagagacagGGTATACCCTCAAGTAGCACTAGTAGTAGTTGGCGCTTGTTACTTAGCGGCAGTGTGTGCTTGCGTGTGGAGTATAGTTAACTGGTAATACGACTCACTAAAGGAGACACTATATGTTTCGACTTCATTACAAC‑AAAGCGTTAAGAATTTCACGGTTCGCCGTGCTGACCGTTCAATCGTATGTGCGAGCGAGCGCCGAGCTAAGATACCTCTTATTGGTAACACAGTTCCTTTGGCACCGAGCGTCCACATCATTATCACCCGTGGTGACTTTGAGAA                                                                                                                             <  1:2960/279‑1`  
                                                                                                                                                                                                                                                              `cgtgggctcggagatgtgtataagagacaGGAGTATAGTTAACTGGTAATACGACTCACTAAAGGAGACACTATATGTTTCGACTTCATTACAACAAAAGCGTTAAGAATTTCACGGTTCGCCGTGCTGACCGTTCAATCGTATGTGCGAGCGAGCGCCGAGCTAAGATACCTCTTATTGGTAACACAGTTCCTTTGGCACCGAGAGTCCACATAATTATCACCCGTGGTGACTTTGAGAAAGCAATAGACAAGAAACGTCCGGTTCTTAGTGTGGCAGTGACCCGCTTCCCGTTCATCCG                                                                 <  1:440283/272‑1`  
                                                                                                                                                                                                                                                                                        `gGTGGAGTATAGTTAACTGGTAATACGACTCACTAAAGGAGACACTATATGTTTCGACTTCATTACAACAAAAGCGTTAAGAATTTCACGGTTCGCCGTGCTGACCGTTCAATCGTATGTGCGAGCGAGCGCCGAGCTAAGATACCTCTTATTGGTAACACAGTTCCTTTGGCACCGAGCGTCCACATCATTATCACCCGTGGTGACTTTGAGAAAGCAATAGACAAGAAACGTCCTGTTCTTAGTGTGGCAGTGACCCGCTTCCCGTTCGTCCGTCTGTTACTCAAACGAATCAAGGAGG                                       <  1:185583/300‑1`  
                                                                                                                                                                                                                                                                                              `GTATAGTTAACTGGTAATACGACTCACTAAAGGAGACACTATATGTTTCGACTTCATTACAACAAAAGCGTTAAGAATTTCACGGTTCGCCGTGCTGACCGTTCAATCGTATGTGCGAGAGAGCGCCGAGCTAAGATACCTCTTATTGGTAACACAGTTCCTTTGGCACCGAGCGTCCACATCATTATCACCCGTGGTGACTTTGAGAACGCAATAGACAAGAAACGTCCGGTTCTTAGTGTGGCAGTGACCCGCTTCCCGTTCGTCCGTCTGTTACTCAAACGAATCAAGGAGGTGTTCT                                 >  1:316922/1‑301`  
                                                                                                                                                                                                                                                                                                  `gagatgtttataagagacagCACTAAAGGAGACACTATATGTTTCGACTTCATTACAACAAAAGCGTTAAGAATTTCACGGTTCGCCGTGCTGACCGTTCAATCGTATGTGCGAGCGAGCGCCGAGCTAAGATACCTATTATTGGTAACACAGTTCCTTTGGCACCGAGCGTCCACATCATTATCACCCGTGGTGACTTTGAGAAAGCAATAGACAAGAAACGTCCGGTTCTTAGTGTGGCAGTGACCCGCTTCCCGTTCGTCCGTCTGTTACTCAAACGAATCAAGGAGGTGTTCTGATG                             <  1:493529/281‑1`  
                                                                                                                                                                                                                                                                                                  `ctcggagatgtgtataagagacagAAAGGAGACACTATATGTTTCGACTTCATTACAACAAAAGCGTTAAGAATTTCACGGTTCGCCGTGCTGACCGTTCAATCGTATGTGCGAGCGAGCGCCGAGCTAAGATACCTCTTATTGGTAACACAGTTCCTTTGGCACCGAGCGTCCACATCATTATCACCCGTGGTGACTTTGAGAAAGCAATAGACAAGAAACGTCCGGTTCTTAGTGTGGCAGTGACCCGCTTCCCGTTCGTCCGTCTGTTACTCAAACGAATCAAGGAGGTGTTCTGATG                             <  1:158294/277‑1`  
                                                                                                                                                                                                                                                                                                      `tggttaaaagagacagcaaTAAAGGAGACACTATATGTTTCGACTTCATTACAACAAAAGCGTTAAGAATTTCACGGTTCGCCGTGCTGACCGTTCAATCGTATGTGCGAGCGATCGCCGAGCTAAGATACCTCTTATTGGTAACACAGTTCCTTTGGCACCGAGCGTCCACATCATTATCACCCGTGGTGACTTTGAGAAAGCAATAGACAAGAAACGTCCGGTTCTTAGTGTGGCAGTGACCCGCTTCCCGTTCGTCCGTATGTTACTCAAACGAATCAAGGAGGTGTTCTGATGGGAC                         <  1:190823/282‑1`  
                                                                                                                                                                                                                                                                                                            `TAATATGACTCACTAAAGGAGACACTATATGTTTCGACTTCATTACAACAAAAGCGTTAAGAATTTCACGGTTCGCCGTGCTGACCGTTCAATCGTATGTGCGAGCGAGCGCCGAGCTAAGATACCTCTTATTGGTAACACAGTTCCTTTGGCACCGAGCGTCCACATCATTATCACCCGTGGTGACTTTGAGAAAGCAATAGACAAGAAACGTCCGGTTCTTAGTGTGGCAGTGACCCGCTTCCAGTTCGTCCGTCTGTTACTCAAACGAATCAAGGAGGTGTTCTGATGGGACTGTTAG                   >  1:775518/1‑301`  
                                                                                                                                                                                                                                                                                                            `TAATACGACTCACTAAAGGAGACACTATATGTTTCGACTTCATTACAACAAAATCGTTAAGAATTTCACGGTTCGCCGTGCTGACCGTTCAATCGTATGTGCGAGTGAGCGCCGAGCTAAGATACCTCTTATTGGTAACACAGTTCCTTTGGCACCGAGCGTCCACATCATTATCACCCGTGGTGACTTTGAGAAAGCAATAGACAAGAAACGTCCGGTTCTTAGTGTGGCAGTGACCCGCTTCCCGTTCGTCCGTCTGTTACTCAAACGAATCAAGGAGGTGTTCTGATGGGACTGTTAG                   >  1:55076/1‑301`  
                                                                                                                                                                                                                                                                                                            `TAATACGACTCACTAAAGGAGACACTATATGTTTCGACTTCATTACAACAAAAGCGTTAAGAATTTCACGTTTCGCCGTGCTGACCGTTCAATCGTATGTGCGAGCGAGCGCCGAGCTAAGATATCTCTTATTGGCAATACAGTTCCTTTGGCACCGAGCGTCCACATCATTATTACCAGCGGTGACTTTGAGAATGCAATAGATAAGACACGTCCGGTTCTTAGTGTGGCAGTGACCTGCCTTCCGTTCGTCCGTCCGTTACTCAAACGAATCACGGctgtctcttatacacatcttgac                   >  1:522375/1‑278`  
                                                                                                                                                                                                                                                                                                            `TAATACGACTCACTAAAGGAGACACTATATGTTTCGACTTCATTACAACAAAAGCGTTAAGAATTTCACGGTTCGCCGTGCTGACCGTTCAATCGTATGTGCGAGCTAGCGCCGAGCTAAGATACCTCTTATTGGTAACACAGTTCCTTTGGCACCGAGCGTCCACATCATTATCACCCGTGGTGACTTTGAGAAAGCAATAGACAAGAAACGTCCGGTTCTTAGTGTGGCAGTGACCCGCTTCCCGTTCGTCCGTCTGTTACTCTAACGAATCAAGGAGGTGTTCTGATGGGACTGTTAG                   >  1:125657/1‑301`  
                                                                                                                                                                                                                                                                                                            `TAATACGACTCACTAAAGGAGACACTATATGTTTCGACTTCATTACAACAAAAGCGTTAAGAATTTCACGGTTCGCCGTGCTGACCGTTCAATCGTATGTGCGAGCGAGCGCCGAGCTAAGATACCTCTTATTGGTAACACAGTTCCTTTGGCACCGAGCGTCCACATCATTATCACCCGTGGTGACTTTGAGAAAGCAATAGACAAGAAACGTCCGGTTCTTAGTGTGGCAGTGACCCGCTTCCCGTTCGTCCGTCTGTTACTCAAACGAATCAAGGctgtctcttatacacatctcgag                   >  1:522397/1‑278`  
                                                                                                                                                                                                                                                                                                            `TAATACGACTCACTAAAGGAGACACTATATGTTTCGACTTCATTACAACAAAAGCGTTAAGAATTTCACGGTTCGCCGTGCTGACCGTTCAATCGTATGTGCGAGCGAGCGCCGAGCTAAGATACCTCTTATTGGTAACACAGTTCCTTTGGCACCGAGCGTCCACATCATTATCACCCGTGGTGACTTTGAGAAAGCAATAGACAAGAAACGTCCGGTTCTTAGTGTGGCAGTGACCCGCTTCCCGTTCGTCCGTCTGTTACTCAAACGAATCAAGGActgtctcttatacacatctccc                   >  1:243301/1‑279`  
                                                                                                                                                                                                                                                                                                            `TAATACGACTCACTAAAGGAGACACTATATGTTTCGACTTCATTACAACAAAAGCGTTAAGAATTTCACGGTTCGCCGTGCTGACCGTTCAATCGTATGTGCGAGCGAGCGCCGAGCTAAGATACCTCTTATTGGTAACACAGTTCCTTTGGCACCGAGCGTCCACATCATTATCACCCGTGGTGACTTTGAGAAAGCAATAGACAAGAAACGTCCGGTTCTTAGTGTGGCAGTGACCCGCTTCCCGTTCGTCCGTCTGTTACTCAAACGAATCAAGGAGGTGTTCTGATGGGACctgtct                   >  1:718470/1‑295`  
                                                                                                                                                                                                                                                                                                            `TAATACGACTCACTAAAGGAGACACTATATGTTTCGACTTCATTACAACAAAAGCGTTAAGAATTTCACGGTTCGCCGTGCTGACCGTTCAATCGTATGTGCGAGCGAGCGCCGAGCTAAGATACCTCTTATTGGTAACACAGTTCCTTTGGCACCGAGCGTCCACATCATTATCACCCGTGGTGACTTTGAGAAAGCAATAGACAAGAAACGTCCGGTTCTTAGTGTGGCAGTGACCCGCTTCCCGTTCGTCCGTCTGTTACTCAAACGAATCAAGGAGGTGTTCTGATGGGACTGTTAG                   <  1:313142/301‑1`  
                                                                                                                                                                                                                                                                                                            `TAATACGACTCACTAAAGGAGACACTATATGTTTCGACTTCATTACAACAAAAGCGTTAAGAATTTCACGGTTCGCCGTGCTGACCGTTCAATCGTATGTGCGAGCGAGCGCCGAGCTAAGATACATCTTATTGGTAACACAGTTCCTTTGGCACCGAGCGTCCACATCATTATCACCCGTGGTGACTTTGAGAAAGCAATAGACAAGAAACGTCCGGTTCTTAGTGTGGCAGTGACCCGCTTCCCGTTCGTCCGTCTGTTACTCAAACGAATCAAGGAGGTGTTCTGATGGGACTGTTAG                   >  1:703126/1‑301`  
                                                                                                                                                                                                                                                                                                             `gatgtgtataagagacaGAGACACTATATGTTTCGACTTCATTACAACAAAAGCGTTAAGAATTTCACGGTTCGCCGTGCTGACCGTTCAATCGTATGTGCGAGCGAGCGCCGAGCTAAGATACCTCTTATTGGTAACACAGTTCCTTTGGCACCGAGCGTCCACATCATTATCACCCGTGGTGACTTTGAGAAAGCAATAGACAAGAAACGTCCGGTTCTTAGTGTGGCAGTGACCCGCTTCCCGTTCGTACGTCTGTTACTCAAACGAATCAAGGAGGTGTTCTGATGGGACTGTTAGA                  <  1:593249/284‑1`  
                                                                                                                                                                                                                                                                                                             `aaaACGACTCACTAAAGGAGACACTATATGTTTCGACTTCATTACAACAAAAGCGTTAAGAATTTCACGGTTCGCCGTGCTGACCGTTCAATCGTATGTGCGAGCGAGCGCCGAGCTAAGATACCTCTTATTGGTAACACAGTTCATTTGGCACCGAGCGTCCACATCATTATCACCCGTGGTGACTTTGAGAAAGCAATAGACAAGAAACGTCCGGTTCTTAGTGTGGCAGTGACCCGATTCCCGTTCGTCCGTCTGTTACTCAAACGAATCAAGGAGGTGTTCTGATGGGACTGTTAGA                  <  1:753864/298‑1`  
                                                                                                                                                                                                                                                                                                             `AATACGACTCACTAAAGGAGACACTATATGTTTCGACTTCATTACAACAAAAGCGTTAAGAATTTCACGGTTCGCCGTGCTGACCGTTCAATCGTATGTGCGAGCGAGCGCCGAGCTAAGATACCTCTTATTGGTAACACAGTTCCTTTGGCACCGAGCGTCCACATCATTATCACCCGTGGTGACTTTGAGAAAGCAATAGACAAGAAACGTCCGGTTCTTAGTGTGGCAGTGACCCGCTTCCCGTTCGTCCGTCTGTTACTCAAACGAATCAAGGAGGTGTTCTGATGGGACTGTTAGA                  <  1:177010/301‑1`  
                                                                                                                                                                                                                                                                                                              `agagacagCACTAAAGGAGACACTATATGTTTCGACTTCATTACAACAAAAGCGTTAAGAATTTCACGGTTCGCCGTGCTGACCGTTCAATCGTATGTGCGAGCGAGCGCCGAGCTAAGATACCTATTATTGGTAACACAGTTCCTTTGGCACAGAGCGTCCACATCATTATCACCAGTGGTGACTTTGAGAAAGCAATAGACAAGAAACGTCCGGTTCTTAGTGTGGCAGTGACCCGCTTCCCGTTCGTCCGTCTGTTACTCAAACGAATCAAGGAGGTGTTCTGATGGGACTGATAGAT                 <  1:551445/293‑1`  
                                                                                                                                                                                                                                                                                                              `ATACGACTCACTAAAGGAGACACTATATGTTTCGACTTCATTACAACAAAAGCGTTAAGAATTTCACGGTTCGCCGTGCTGACCGTTCAATCGTATGTGCGAGCGAGCGCCGAGCTAAGATACCTCTTATTGGTAACACAGTTCCTTTGGCACCGAGCGTCCACATCATTATCACCCGTGGTGACTTTGAGAAAGCAATAGACAAGAAACGTCCGGTTCTTAGTGTGGCAGTGACCCGCTTCCCGTTCGTCCGTCTGTTACTCAAACGAATCAAGGAGGTGTTCTGATGGGACTGTTAGAT                 <  1:598807/301‑1`  
                                                                                                                                                                                                                                                                                                              `ATACGACTCACTAAAGGAGACACTATATGTTTCGACTTCATTACAACAAAAGCGTTAAGAATTTCACGGTTCGCCGTGCTGACCGTTCAATCGTATGTGCGAGCGAGCGCCGAGCTAAGATACCTCTTATTGGTAACACAGTTCCTTTGGCACCGAGCGTCAACATCATTATCACCCGTGGTGACTTTGAGAAAGCAATAGACAAGAAACGTCCGGTTCTTAGTGTGGCAGTGACCCGCTTCCCGTTCGTCCGTCTGTTACTCAAACGAATCAAGGAGGTGTTCTGATGGGACTGTTAGAT                 <  1:96501/301‑1`  
                                                                                                                                                                                                                                                                                                                `gCGACTCACTAAAGGAGACACTATATGTTTCGACTTCATTACAACAAAAGCGTTAAGAATTTCACGGTTCGCCGTGCTGACCGTTCAATCGTATGTGCGAGCGAGCGCCGAGCTAAGATACCTCTTATTGGTAACACAGTTCCTTTGGCACCGAGCGTCCACATCATTATCACCCGTGGTGACTTTGAGAAAGCAATAGACAAGAAACGTCCGGTTCTTAGTGTGGCAGTGACCCGCTTCCCGTTAGTCCGTCTGTTACTCAAACGAATCAAGGAGGTGTTCTGATGGGACTGTTAGATGG               <  1:246798/300‑1`  
                                                                                                                                                                                                                                                                                                                `ccgcctaacttaagtgGACACTATATGTTTCGACTTCATTGCAACACATGCGTTCAGAATGTCGCGGTTCGCCGTTCTGACCATTCCATCGTATGTGCGAGCGGGCGCCGAGCTAAGATTCCTCTTTTTGGTAACACAGTTCCTTTGGCACCGAGCGTCCACATCATTATCACCCGTGGTGACTTTTAGAAAGCAATAGACAAGAAACGTCCGGTTCTTAGTGTGGCAGTGACCCGCTTCCCGTTCGTCAGTCTGTTACTCAAACGAATCAAGGAGGTGTTCTGATGGGACTGTTAGATGG               <  1:40072/285‑1`  
                                                                                                                                                                                                                                                                                                                `cCGACTCACTAAAGGAGACACTATATGTTTCGACTTCATTACAACAAAAGCGTTAAGAATTTCACGGTTCGCCGTGCTTACCGTTCAATCGTATGTGCGAGCGAGCGCCGAGCTAAGATACCTCTTATTGGTAACACAGTTCCTTTGGCACCGAGCGTCCACATCATTATCACCCGTGGTGACTTTGAGAAAGCAATAGACAAGAAACGTCCGGTTCTTAGTGTGGCAGTGACCCGCTTCCCGTTCGTCCGTCTGTTACTCAAACGAATCAAGGAGGTGTTCTGATGGGACTGTTAGATGG               <  1:362716/300‑1`  
                                                                                                                                                                                                                                                                                                                `aaagagacagAAAGGAGACACTATATGTTTCGACTTCATTACAACAAAAGCGTTAAGAATTTCACGGTTCGCCGTGCTGACCGTTCAATCGTATGTGCGAGCGAGCGCCGAGCTAAGATACCTCTTATTGGTAACACAGTTCCTTTGGCACCGAGCGTCCACATCATTATCACCCGTGGTGACTTTGAGAAAGCAATAGACAAGAAACGTCCGGTTCTTAGTGTGGCAGTGACCCTCTTCCCGTTCGTCCGTCTGTTACTCAAACGAATCAAGGAGGTGTTCTGATGGGACTGTTAGATGG               <  1:677692/291‑1`  
                                                                                                                                                                                                                                                                                                                `ACGACTCACTAAAGGAGACACTATATGTTTTGACTTCATTACAACAAAAGCGTTAAGAATTTCACGGTTCGCCGTGCTGACCGTTCAATCGTATGTGCGAGCGAGCGCCGAGCTAAGATACCTCTTATTGGTAACACAGTTCCTTTGGCACCGAGCGTCCACATCATTATCACCCGTGGTGACTTTGAGAAAGCAATAGACAAGAAACGTCCGGTTCTTAGTGTGGCAGTGACCCGCTTCCCGTTCGTCCGTCTGTTACTCAAACGAATCAAGGAGGTGTTCTGATGGGACTGTTAGATGG               <  1:115804/301‑1`  
                                                                                                                                                                                                                                                                                                                `ACGACTCACTAAAGGAGACACTATATGTTTCGACTTCATTACAACAAAAGCGTTAAGAATTTCACGGTTCGCCGTGCTTACCGTTCAATCGTATGTGCGAGCGAGCGCCGAGCTAAGATACCTCTTATTGGTAACACAGTTCCTTTGGCACCGAGCGTCCACATCATTATCACCCGTGGTGACTTTGAGAAAGCAATAGACAAGAAACGTCCGGTTCTTAGTGTGGCAGTGACCCGCTTCCCGTTCGTCCGTCTGTTACTCAAACGAATCAAGGAGGTGTTCTGATGGGACTGTTAGATGG               <  1:362711/301‑1`  
                                                                                                                                                                                                                                                                                                                `ACGACTCACTAAAGGAGACACTATATGTTTCGACTTCATTACAACAAAAGCGTTAAGAATTTCACGGTTCGCCGTGCTGACCGTTCAATCGTATGTGCGAGCGAGCGCCGAGCTAAGATACCTCTTATTGGTAACACAGTTCCTTTGGCACCGAGCGTCCACATCATTATCACCCGTGGTGACTTTGAGAAAGCAATAGACAAGAAACGTCCGGTTCTTAGTGTGGCAGTGACCCGCTTCCCGTTCGTCCGTCTGTTACTCAAACGAATCAAGGAGGTGTTCTGATGGGACTGTTAGATGG               <  1:179087/301‑1`  
                                                                                                                                                                                                                                                                                                                `ACGACTCACTAAAGGAGACACTATATGTTTCGACTTCATTACAACAAAAGCGTTAAGAATTTCACGGTTCGCCGTGCTGACCGTTCAATCGTATGTGCGAGCGAGCGCCGAGCTAAGATACCTCTTATTGGTAACACAGTTCCTTTGGCACCGAGCGTCCACATCATTATCACCCGTGGTGACTTTGAGAAAGCAATAGACAAGAAACGTCCGGTTCTTAGTGTGGCAGTGACCCGCTTCCCGTTCGTCCGTCTGTTACTCAAACGAATCAAGGAGGTGTTATGATGGGACTGTTAGATGG               <  1:436721/301‑1`  
                                                                                                                                                                                                                                                                                                                   `ACTCACTAAAGGAGACACTATATGTTTCGACTTCATTACAACAAAAGCGTTAAGAATTTCACGGTTCGCCGTGCTGACCGTTCAATCGTATGTGCGAGCGAGCGCCGATCTAAGATACCTCTTATTGGTAACACAGTTCCTTTGGCACCGAGCGTCCACATCATCATCACCCGTGGTGACTTTGAGAAAGCAATAGACAAGAAACGTCCTGTTCTTAGTGTGGCAGTGACCCGCTTCCCGTTCGTCCGTCTGTTACTCAAACTAATCAAGGAGGTGTTCTGATGTGACTGTTAGATGGctg            >  1:9174/1‑298`  
                                                                                                                                                                                                                                                                                                                   `ACTCACTAAAGGAGACACTATATGTTTCGACTTCATTACAACAAAAGCGTTAAGAATTTCACGGTTCGCCGTGCTGACCGTTCAATCGTATGTGCGAGCGAGCGCCGAGCTAAGATACCTCTTATTGGTAACACAGTTCCTTTGGCACCGAGCGTCCACATCATTATCACCCGTGGTGACTTTGAGAAAGCAATAGACAAGAAACGTCCGGTTCTTAGTGTGGCAGTGACCCGCTTCCCGTTCGTCCGTCTGTTACTCAAACGAATCAAGGAGGTGTTCTGATGGGACTGTTAGATGGctg            >  1:301604/1‑298`  
                                                                                                                                                                                                                                                                                                                   `ACTCACTAAAGGAGACACTATATGTTTCGACTTCATTACAACAAAAGCGTTAAGAATTTCACGGTTCGCCGTGCTGACCGTTCAATCGTATGTGCGAGCGAGCGCCGAGCTAAGATACCTCTTATTGGTAACACAGTTCCTTTGGCACCGAGCGTCCACATCATTATCACCCGTGGTGACTTTGAGAAAGCAATAGACAAGAAACGTCCGGTTCTTAGTGTGGCAGTGACCCGCTTCCCGTTCGTCCGTCTATTACTCAAACGAATCAAGGAGGTGTTCTGATGGGACTGTTAGATGGTGA            >  1:98167/1‑301`  
                                                                                                                                                                                                                                                                                                                      `CACTAAAGGAGACACTATATGTTTCGACTTCATTACAACAAAAGCGTTAAGAATTTCCCGGTTCGCCGTGCTGACCGTTCAATCTTATGTGCGAGCGAGCGCCGCGCTAAGATACCTCTTATTGGTAACACAGTTCCTTTGGCACCGAGCGTCCACATCATTCTCACCCGTGGTGACTTTGCGAAAGCAATAGACAAGAAACATCCGGTTCTTAGTGTGGCAGTTACCCGCTTCCCGTTCGTCCGTCTGTTACTCACACGAATCAAGGAGGTGTTCTGATGGCACTGTTAGATGGTGAAGC         >  1:586580/1‑301`  
                                                                                                                                                                                                                                                                                                                      `CACTAAAGGAGACACTATATGTTTCGACTTCATTACAACAAAAGCGTTAAGAATTTCACGGTTCGCCGTGCTGACCGTTCAATCGTATGTGCGAGCGAGCGCCGAGCTAAGATACCTCTTATTGGTAACACAGTTCCTTTGGCACCGAGCGTCCACATCATTATCACCCGTGGTGACTTTGAGAAAGCAATAGACAAGAAACGTCCGGTTCTTAGTGTGGCAGTGACCCGCTTCCCGTTCGTCCGTCTGTTACTCAAACGAATCAAGGAGGTGctgtctcttatacacatctccgagcccc         >  1:51562/1‑273`  
                                                                                                                                                                                                                                                                                                                      `CACTAAAGGAGACACTATATGTTTCGACTTCATTACAACAAAAGCGTTAAGAATTTCACGGTTCGCCGTGCTGACCGTTCAATCGTATGTGCGAGCGAGCGCCGAGCTAAGATACCTCTTATTGGTAACACAGTTCCTTTGGCACCGAGCGTCCACATCATTATCACCAGTGGTGACTTTGAGAAAGCAATAGACAAGAAACGTCCGGTTCTTAGTGTGGCAGTGACCCGCTTCCCGTTCGTCCGTCTGTTACTCAAACGAATCAAGGAGGTGTTCTGATGGGACTGTTAGATGGTGAAGC         >  1:128848/1‑301`  
                                                                                                                                                                                                                                                                                                                      `CACTAAAGGAGACACTATATGTTTCGACTTCATTACAACAAAAGCGTTAAGAATTTCACGGTTCGCCGTGCTGACCGTTCAATCGTATGTGCGAGCGAGCGCCGAGCTAAGATACCTCTTATTGGTAACACAGTTCCTTTGGCACCGAGCGTCCACATAATTATCACCCGTGGTGACTTTGAGAAAGCAATAGACAAGAAACGTCCGGTTCTTAGTGTGGCAGTGACCCGCTTCCCGTTCGTCCGTCTGTTACTCAAACGAATCAAGGAGGTGTTCTGATGctgtctcttatacacatctc         >  1:761537/1‑281`  
                                                                                                                                                                                                                                                                                                                      `CACTAAAGGAGACACTATATGTTTCGACTTCATTACAACAAAAGCGTTAAGAATTTCACGGTTCGCCGTGATGACCGTTCAATCGTATGCGCGAGCGAGCGCCGAGCTAAGATACCTCTTATTGGTAACACAGTTCCTTTGGCACCGAGCGTCCACATCATTATCACACGTGGTGACTTTGAGAAAGCAATAGACAAGAAACGTCCGGTTCTTAGTGTGGCAGTGACCCGATTCCCGTTCGTCCGTCTGTTACTCAAACGAATCAAGGAGGTGTTCTGATGGGACctgtctcttatacaca         >  1:438054/1‑285`  
                                                                                                                                                                                                                                                                                                                      `CACTAAAGGAGACACTATATGTTTCGACTTCATTACAACAAAAGCGTTAAGAATTTCAAGGTTCGCCGTGCTGACCGTTCAATCGTATGTGCGAGCGAGCGCCGAGCTAAGATACCGCTTATTGGTAACACAGTTCCATTGGCACGGTGCGTCCACATCATTCTCACCCGTGGTGACTTTCAGCAAGCAATAGACAAGAAACGTCCGGATCTTAGTGTGTCATTGACTCGCTTCACGTTCGTCCATCTGTTACTTTAACTAATCAAGGAGGGGCTCTGTTGTGACTGTTAGATGGTGctgt         >  1:515342/1‑297`  
                                                                                                                                                                                                                                                                                                                      `CACTAAAGGAGACACTATATGTTTCGACTTAATTGCAACGAAAGCGTTCAGAATTTCACGGTTCGCCGTGCTGACCGTTCAATCGTATGTGCGAGCGAGCGCCTAGCTAAGATACCTCTTATTGGTAACACAGTTCCTTTGGCACCGAGCGTCCACATCATTATCACCCGTGGTGACTTTGAGAACGCAATAGACAAGAAACGTCCGGTTCTTAGTGTGTCAGTGACCCGCTTCCCGTTCGTCCGTCTGTTACTCACACGAATCAAGGAGGctgtctcttatacacatctccgagcccacg         >  1:587255/1‑271`  
                                                                                                                                                                                                                                                                                                                       `gCTAAAGGAGACACTATATGTTTCGACTTCATTACAACAAAAGCGTTAAGAATTTCACGGTTCGCCGTGCTGACCGTTCAATCGTATGTGCGAGCGAGCGCCGAGCTAAGATACCTCTTATTGGTAACACAGTTCCTTTGGCACCGAGCGTCCACATCATTATCACCCGTGGTGACTTTGAGAAAGCAATAGACAAGAAACGTCCGGTTCTTAGTGTGGCAGTGACCCGCTTCCCGTTCGTCCGTCTGTTACTCAAACGAATCAAGGAGGTGTTCTGATGGGACTGTTAGATGGTGAAGCC        <  1:228184/300‑1`  
                                                                                                                                                                                                                                                                                                                       `cCTAAAGGAGAAACTATATTTTTCTACTTCATTACAACAAAAGCGTGAATAATTGCACGGTTCGCCGGGCTGACCGTTCAATCGTATGTGCGAGCGAGCGCCGAGCTAAGATACCTCTTATTGGTAACACAGTTCCTTTGGCACTGAGCGTCCACATCATTATCACCCGTGGTGACTTTGAGAAAGCAATAGACAAGAAACGTCCGGTTCTTAGTGTGGCAGTGACACGCTTCCCGTTCGTCCGTCTGTTACTCAAACGAATCAAGGAGGTGTTCTGATGGGACTGTTAGATGGTGAAGCC        <  1:234932/300‑1`  
                                                                                                                                                                                                                                                                                                                       `acaAAAGAAGACACTATATGTTTCGACTTCATTACAACAAAAGCGTTAAGAATTTCACGGTTCGCCGTGCTGACCGTTCAATCGTATGTGCGAGCGAGCGCCGAGCTAAGATACCTCTTATTGGTAACACAGTTCCTTTGGCACCGAGCGTCCACATCAATATCACCCGTGGTGACTTTGAGAAAGCAATAGACAAGAAACGTCCGGTTCTTAGTGTTGCAGTGACCCGATTCCCGTTCGTCCGTCTGTTACTCAAACGAATCAAGGAGGTGTTCTGATGGGACTGTTAGATGGTGAAGCC        <  1:537921/298‑1`  
                                                                                                                                                                                                                                                                                                                       `ACTAAAGGAGACACTATATGTTTCGACTTCATTACAACAAAAGCGTTAAGAATTTCACGGTTCGCCGTGCTGACCGTTCAATCGTATGTGCGAGCGAGCGCCGAGCTAAGATACCTCTTATTGGTAACACAGTTCCTTTGGCACCGAGCGTCCACATCATTATCACCCGTGGTGACTTTGAGAAAGCAATAGACAAGAAACGTCTGGTTCTTAGTGTGGCAGTGACCCGCTTCCCGTTCGTCCGTCTGTTACTCCAACGAATCAAGGAGGTGTTCTGATGGGACTATTAGActgtctcttt        >  1:358427/1‑291`  
                                                                                                                                                                                                                                                                                                                       `ACTAAAGGAGACACTATATGTTTCGACTTCATTACAACAAAAGCGTTAAGAATTTCACGGTTCGCCGTGCTGACCGTTCAATCGTATGTGCGAGCGAGCGCCGAGCTAAGATACCTCTTATTGGTAACACAGTTCCTTTGGCACCGAGCGTCCACATCATTATCACCCGTGGTGACTTTGAGAAAGCAATAGACAAGAAACGTCCGGTTCTTAGTGTGGCAGTGACCCGCTTCCCGTTCGTCCGTCTGTTACTCAAACGAATCAAGGAGGTGTTCTGATGGGACTGTTAGATGGTGAAGCC        <  1:429803/301‑1`  
                                                                                                                                                                                                                                                                                                                       `ACTAAAGGAGACACTATATGTTTCGACTTAATTACAACAAAAGCGTTAAGAATTTCACGGTTCGCCGTGCTGACCGTTCAATCGTATGTGCGAGCGAGCGCCGAGCTAAGATACCTCTTATTGGTAACACAGTTCCTTTGGCACCGAGCGTCAACATCATTATCACCCGTGGTGACTTTGAGAAAGCAATAGACAAGAAACGTCCGGTTCTTAGTGTGGCAGTGACACGCTTCCCGTTCGTCCGTCTGTTACTCAAACGAATCAAGGAGGTGTTCTGATGGGACTGTTAGATctgtctctt        >  1:482693/1‑292`  
                                                                                                                                                                                                                                                                                                                        `CTACAGGATACACTATATGTTTCGACTTCATTACAACAAAAGCGTTAAGAATTTCACGGTTCGCCGTGCTGACCGTTCAATCGTATGTGCGAGCGAGCGCCGAGCTAAGATACCTCTTATTGGTAACACAGTTCCTTTGGCACCGAGCGTCCACATCATTATCACCCGTGGTGACTTTGAGAAAGCAATAGACAAGAAACGTCCGGTTCTTAGTGTGGCAGTGACCCGCTTCCCGTTCGTCCGTCTGTTACTCAAACGAATCAAGGAGGTGTTCTGATGGGACTGTTAGATGGTGAAGCCT       >  1:157804/1‑301`  
                                                                                                                                                                                                                                                                                                                        `CTAAAGTAGACACTATATGTTTCGACTTCATTACAACAAAAGCGTTAAGAATTTCACGGTTCGCCGTGCTGACCGTTCAATCGTATGTGCGAGCGAGCGCCGAGCTAAGATACCTCTTATTGGTAACACAGTTCCTTTGGCACCGAGCGTCCACATCATTATCACCCGTGGTGACTTTGAGAAAGCAATAGACAAGAAACGTCCGGTTCTTAGTGTGGCAGTGACCCGCTTCCCGTTCGTCCGTCTGTTACTCAAACGAATCAAGGAGGTGTTCTGATGGGACTGTTAGATGGTGAAGCCT       >  1:180947/1‑301`  
                                                                                                                                                                                                                                                                                                                        `CTAAAGGATACACTATATGTTTCGACTTCATTACAACAAAAGCGTTAAGAATTTCACGGTTCGCCGTGCTGACCGTTCAATCGTATGTGCGAGCTAGCGCCGAGCTAAGATACCTCTTATTGGTAACACAGTTCCTTTGGCACCGAGCGTCCACATCATTATCACCCGTGGTGACTTTGAGAAAGCAATAGACAAGAAACGTCCGGTTCTTAGTGTGGCAGTGACCCGCTTCCCGTTCGTCCGTCTGTTACTCAAACGAATCAAGGAGGTGTTCTGATGGGctgtctcttatacacatctc       >  1:376181/1‑281`  
                                                                                                                                                                                                                                                                                                                        `CTAAAGGAGACACTATATGTTTCGACTTCATTACAACAAAAGCGTTAAGAATTTCACGGTTCGCCGTGCTGACCGTTCAATCGTATGTGCGAGCGAGCGCTGAGCTAAGATACCTCTTATTGGTAACACAGTTCCTTTGGCACCGAGCGTCCACATCATTATCACCCGTGGTGACTTTGAGAAAGCAATAGACAAGAAACGTCCGGTTCTTAGTGTGGCAGTGACCCGCTTCCCGTTCGTCCGTCTGTTACTCAAACGAATCAAGGAGGTGTTCTGATGGGACTGTTAGATGGTGAAGCCT       >  1:404769/1‑301`  
                                                                                                                                                                                                                                                                                                                        `CTAAAGGAGACACTATATGTTTCGACTTCATTACAACAAAAGCGTTAAGAATTTCACGGTTCGCCGTGCTGACCGTTCAATCGTATGTGCGAGCGAGCGCCGAGCTAAGATACCTCTTATTGGTAACACAGTTCCTTTGGCACCGAGCGTCCACATCATTATCACCCGTGGTGACTTTGAGAAAGCAATAGACAAGAAACGTCCGGTTCTTAGTGTGGCAGTGACCCGCTTCCCGTTCGTCCGTCTGTTACTCAAACGAATCAAGGAGGTGTTCTGATGGGACTGTTAGATGGTGAAGCCT       >  1:376076/1‑301`  
                                                                                                                                                                                                                                                                                                                        `CTAAAGGAGACACTATATGTTTCGACTTCATTACAACAAAAGCGTTAAGAATTTCACGGTTCGCCGTGCTGACCGTTCAATCGTATGTGCGAGCGAGCGCCGAGCTAAGATACCTCTTATTGGTAACACAGTTCCTTTGGCACCGAGCGTCCACATCATTATCACCCGTGGTGACTTTGAGAAAGCAATAGACAAGAAACGTCCGGTTCTTAGTGTGGCAGTGACCCGCTTCCCGTTCGTCCGTCTGTTACTCAAACGAATCAAGGAGGTGTTCTGATGGGACTGTTAGATGGTGAAGCCT       >  1:658789/1‑301`  
                                                                                                                                                                                                                                                                                                                            `caGAGACACTATATGTTTCGACTTCATTACAACAAAAGCGTTAAGAATTTCACGGTTCGCCGTGCTGACCGTTCAATCGTATGTGCGAGCGAGCGCCGAGCTAAGATACCTCTTATTGGTAACACAGTTCCTTTGGCACCGAGCGTCCACATCATTATCACCCGTGGCGACTTTGAGAAAGCAATAGACAAGAAACGTCCGGTTCTTAGTGTGGCAGTGACCCGCTTCCCGTTCGTCCGTCTGTTACTCAAACGAATCAAGGAGGTGTTCTGATGGGACTGTTAGATGGTGAAGCCTGGGA   <  1:733740/299‑1`  
                                                                                                                                                                                                                                                                                                                            `AGGAGACACTATATGTTTCGACTTCATTACAACAAAAGCGTTAAGAATTTCACGGTTCGCCGTGCTGACCGTTCAATCGTATGTGCGAGCGAGCGCTGAGCTAAGATACCTCTTATTGGTAACACAGTTCCTTTGGCACCGAGCGTCCACATCATTATCACTCGTGGTGACTTTGAGAAAGCAATAGACAAGAAACGTCCGGTTCTTAGTGTGGCAGTGACCCGCTTCCCGTTCGTCCGTCTGTTACTCAAACGAATCAATGAGGTGTTCTGATGGGACTGTTAGATGGTGAAGCCTGGGA   >  1:110285/1‑301`  
                                                                                                                                                                                                                                                                                                                            `AGGAGACACTATATGTTTCGACTTCATTACAACAAAAGCGTTAAGAATTTCACGGTTCGCCGTGCTGACCGTTCAATCGTATGTGCGAGCGAGCGCCGAGCTAAGATACCTCTTATTGGTAACACAGTTCCTTTGGCACCGAGCGTCCACATCATTATCACCCGTGGTGACTTTGAGAAAGCAATAGACAAGAAACGTCCGGTTCTTAGTGTGGCAGTGACCCGCTTCCCGTTCGTCCGTCTGTTACTCAAACGAATCAAGGAGGTGTTCTGATGGGACTGTTAGATGGTGAAGCCTGGGA   >  1:257979/1‑301`  
                                                                                                                                                                                                                                                                                                                            `AGGAGACACTATATGTTTCGACTTCATTACAACAAAAGCGTTAAGAATTTCACGGTTCGCCGTGCTGACCGTTCAATCGTATGTGCGAGCGAGCGCCGAGCTAAGATACCTATTATTGGTAACACAGTTCCTTTGGCACCGAGCGTCCACATCATTATCACCCGTGGTGACTTTGAGAAAGCAATAGACAAGAAACGTCCGGTTCTTAGTGTGGCAGTGACCCGCTTCCCGTTCGTCCGTCTGTTACTCAAACGAATCAAGGAGGTGTTCTGATGGGACTGTTAGATGGTGAAGCCTGGGA   <  1:729894/301‑1`  
                                                                                                                                                                                                                                                                                                                            `AGGAGACACTAAATGTTTCGACTTCATTACAACAAAAGCGTTAAGAATTTCACGGTTCGCCGTGCTGACCGTTCAATCGTATGTGCGAGCGAGCGCCGAGCTAAGATACCTCTTATTGGTAACACAGTTCCTTTGGCACCGAGCGTCCACATCATTATCACCCGTGGTGACTTTGAGAAAGCAATAGACAAGAAACGTCCGGTTCTTAGTGTGGAAGTGACCCGCTTCCCGTTCGTCCGTCTGTTACTCAAACGAATCAAGGAGGTGTTCTGATGGGACTGTTAGATGGTGAAGCCTGGGA   <  1:392826/301‑1`  
                                                                                                                                                                                                                                                                                                                             `aGAGACACTATATGTTTCGACTTCATTACAACAAAAGCGTTAAGAATTTCACGGTTCGCCGTGCTGACCGTTCAATCGTATGTGCGAGCGAGCGCCGAGCTAAGATACCTCTTATTGGTAACACAGTTCCTTTGGCACCGAGCGTCCACATCATTATCACCCGTGGTGACTTTGAGAAAGCAATAGACAAGAAACGTCAGGTTCTTAGTGTGGCAGTGACCCGCTTCCCGTTCGTCCGTCTGTTACTCAAACGAATCAAGGAGGTGTTCTGATGGGACTGTTAGATGGTGAAGCCTGGGAA  <  1:365205/300‑1`  
                                                                                                                                                                                                                                                                                                                             `aGAGACACTATATGTTTCGACTTAATTACAACAAAAGCGTTAAGAATTTCACGGTTCGCCGTGCTGACCGTTCAATCGTATGTGCGAGCGAGCGCCGAGCTAAGATACCTCTTATTGGTAACACAGTTCCTTTGGCACCGAGCGTCCACATCATTATCACCAGTGGTGACTTTGAGAAAGCAATAGACAAGAAACGTCCGGTTCTTAGTGTGGCAGTGACCCGCTTCCCGTTCGTCCGTCTGTTACTCAAACGAATAAAGGAGGTGTTCTGATGGGACTGTTAGATGGTGAAGCCTGGGAA  <  1:730791/300‑1`  
                                                                                                                                                                                                                                                                                                                             `GGAGACACTATATGTTTCGACTTCATTACAACAAAAGCGTTAAGAATTTCACGGTTCGCCGTGCTGACCGTTCAATCGTATGTGCGAGCGAGCGCCGAGCTAAGATACCTCTTATTTGTAACACAGTTCCTTTGGCACCGAGCGTCCACATCATTATCACCCGTGGTGACTTTGAGAAAGCAATAGACAAGAAACGTCCGGTTCTTAGTGTGGCAGTGACCCGCTTCCCGTTCGTCCGTCTGTTACTCAAACGAATCAAGGAGGTGTTCTGATGGGACTGTTAGATGGTGAAGCCTGGGAA  >  1:111460/1‑301`  
                                                                                                                                                                                                                                                                                                                             `GGAGACACTATATGTTTCGACTTCATTACAACAAAAGCGTTAAGAATTTCACGGTTCGCCGTGCTGACCGTTCAATCGTATGTGCGAGCGAGCGCCGAGCTAAGATACCTATTATTGGTAACACAGTTCCTTTGGCACCGAGCGTCCACATCATTATCACCCGTGGTGACTTTGAGAAAGCAATAGACAAGAAACGTCCGGTTCTTAGTGTGGCAGTGACCCGCTTCCCGTTCGTCCGTCTGTTACTCAAACGAATCAAGGAGGTGTTCTGATactgtctcttatacacatctccgagccc  >  1:35859/1‑273`  

                                                                                                                                                                                                                                                                                                                                                                                                                                                                                                                                                                                                                                            
`TAGCTACATGGAGGAAACACCTGATGGCTCTTTGCGGCACCCATCGTTCGTAATGTTCCGTGGCACCGAGGACAACCCTCAAGAGAAAATGTAATCACACTGGCTCACCTTCGGGTGGGCCTTTCTGCGTTTATAAGGAGACACTTTATGTTTAAGAAGGTTGGTAAATTCCTTGCGGCTTTGGCAGCTATCCTGACGCTTGCGTATATTCTTGCGGTATACCCTCAAGTAGCACTAGTAGTAGTTGGCGCTTGTTACTTAGCGGCAGTGTGTGCTTGCGTGTGGAGTATAGTTAACTGGTAATACGACTCACTAAAGGAG‑‑‑‑‑‑‑‑‑‑‑‑‑‑‑‑‑‑‑‑‑‑‑‑‑‑‑‑‑‑‑‑‑‑‑‑‑‑‑‑‑‑‑‑‑‑‑‑‑‑‑‑‑‑‑‑‑‑‑‑‑‑‑‑‑‑‑‑‑‑‑‑‑‑‑‑‑‑‑‑‑‑‑‑‑‑‑‑‑‑‑‑‑‑‑‑‑‑‑‑‑‑‑‑‑‑‑‑‑‑‑‑‑‑‑‑‑‑‑‑‑‑‑‑‑‑‑‑‑‑‑‑‑‑‑‑‑‑‑‑‑‑‑‑‑‑‑‑‑‑‑‑‑‑‑‑‑‑‑‑‑‑‑‑‑‑‑‑‑‑‑‑‑‑‑‑‑‑‑‑‑‑‑‑‑‑‑‑‑‑‑‑‑‑‑‑‑‑‑‑‑‑‑‑‑‑‑‑‑‑‑‑‑‑‑‑‑‑‑‑‑‑‑‑‑‑‑‑‑‑‑‑‑‑‑‑‑‑‑‑‑‑‑‑‑‑‑‑‑‑‑‑‑‑‑‑‑‑‑‑‑‑‑‑‑‑‑‑‑‑‑‑‑‑‑‑‑‑‑‑‑‑‑‑‑‑‑‑‑‑‑‑‑‑‑‑‑  >  NC_001604.1/7461‑7781`  
`‑‑‑‑‑‑‑‑‑‑‑‑‑‑‑‑‑‑‑‑‑‑‑‑‑‑‑‑‑‑‑‑‑‑‑‑‑‑‑‑‑‑‑‑‑‑‑‑‑‑‑‑‑‑‑‑‑‑‑‑‑‑‑‑‑‑‑‑‑‑‑‑‑‑‑‑‑‑‑‑‑‑‑‑‑‑‑‑‑‑‑‑‑‑‑‑‑‑‑‑‑‑‑‑‑‑‑‑‑‑‑‑‑‑‑‑‑‑‑‑‑‑‑‑‑‑‑‑‑‑‑‑‑‑‑‑‑‑‑‑‑‑‑‑‑‑‑‑‑‑‑‑‑‑‑‑‑‑‑‑‑‑‑‑‑‑‑‑‑‑‑‑‑‑‑‑‑‑‑‑‑‑‑‑‑‑‑‑‑‑‑‑‑‑‑‑‑‑‑‑‑‑‑‑‑‑‑‑‑‑‑‑‑‑‑‑‑‑‑‑‑‑‑‑‑‑‑‑‑‑‑‑‑‑‑‑‑‑‑‑‑‑‑‑‑‑‑‑‑‑‑‑‑‑‑‑‑‑‑‑‑‑‑‑‑‑‑‑‑‑‑‑‑‑‑‑‑‑‑‑‑‑‑‑‑‑‑‑‑‑‑‑‑‑‑‑‑‑‑‑taatacgactcactaaaggagACACTATATGTTTCGACTTCATTACAACAAAAGCGTTAAGAATTTCACGGTTCGCCGTGCTGACCGTTCAATCGTATGTGCGAGCGAGCGCCGAGCTAAGATACCTCTTATTGGTAACACAGTTCCTTTGGCACCGAGCGTCCACATCATTATCACCCGTGGTGACTTTGAGAAAGCAATAGACAAGAAACGTCCGGTTCTTAGTGTGGCAGTGACCCGCTTCCCGTTCGTCCGTCTGTTACTCAAACGAATCAAGGAGGTGTTCTGATGGGACTGTTAGATGGTGAAGCCTGGGAA  >  NC_001604.1/7899‑8195`

  

Alignment Legend

Aligned base mismatch/match (shaded by quality score): `ATCG/ATCG` `< 3 ≤` `ATCG/ATCG` `< 18 ≤` `ATCG/ATCG` `< 29 ≤` `ATCG/ATCG` `< 37 ≤` `ATCG/ATCG` `< 38 ≤` `ATCG/ATCG`

Unaligned base: `atcg`    Masked matching base: `atcg`    Alignment gap: `‑`    Deleted base: `‑`

Reads not counted as support for junction

read\_name Not counted due to insufficient overlap past the breakpoint.

read\_name Not counted due to not crossing MOB target site duplication.
